# Supplementary figures and images for: PSME3 regulates migration and differentiation of myoblasts
Source: Life Sci Alliance. 2025 Jun 19;8(9):e202503208. doi: 10.26508/lsa.202503208 (PMC12179657; doi:10.26508/lsa.202503208)

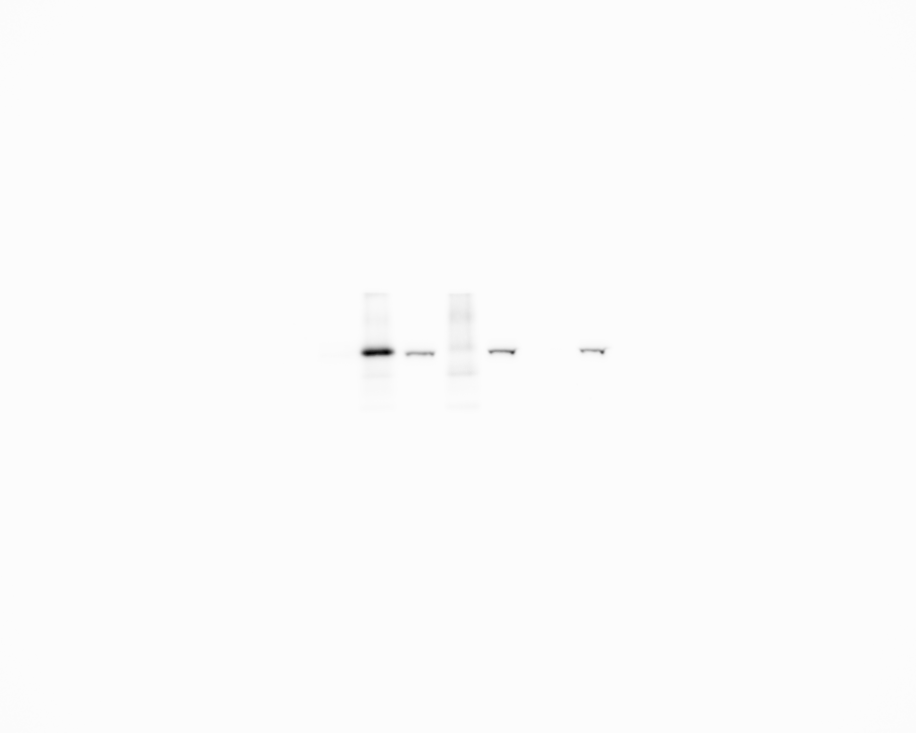

Supplement: Supplementary file 3 [file LSA-2025-03208_SdataF2.zip › Figure_2_Source_Data/PSME3 2023-08-01 15h41m07s(Chemiluminescence).raw16.tif]

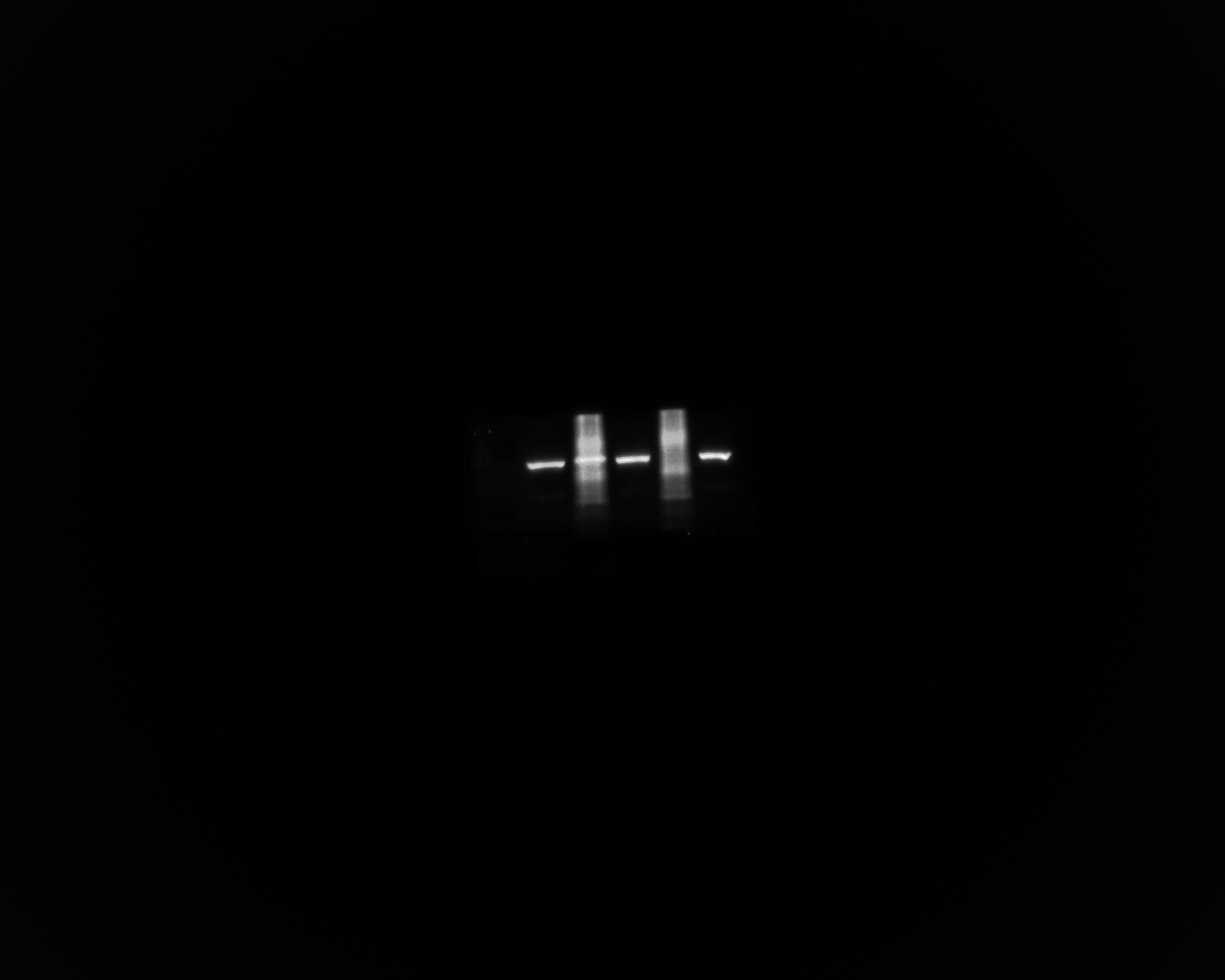

Supplement: Supplementary file 3 [file LSA-2025-03208_SdataF2.zip › Figure_2_Source_Data/rprd1a from e3 ip 2024-01-24 01h40m50s(Chemiluminescence).raw16.tif]

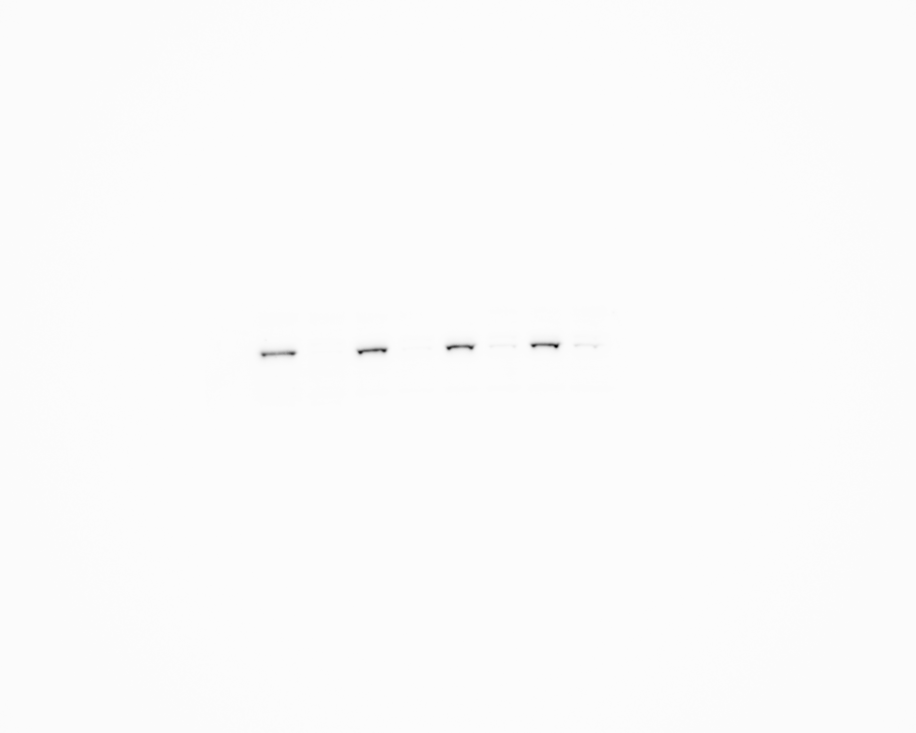

Supplement: Supplementary file 7 [file LSA-2025-03208_SdataF4.zip › Figure_4_Source_Data/psme3 2023-08-11 11h55m45s(Chemiluminescence).raw16.tif]

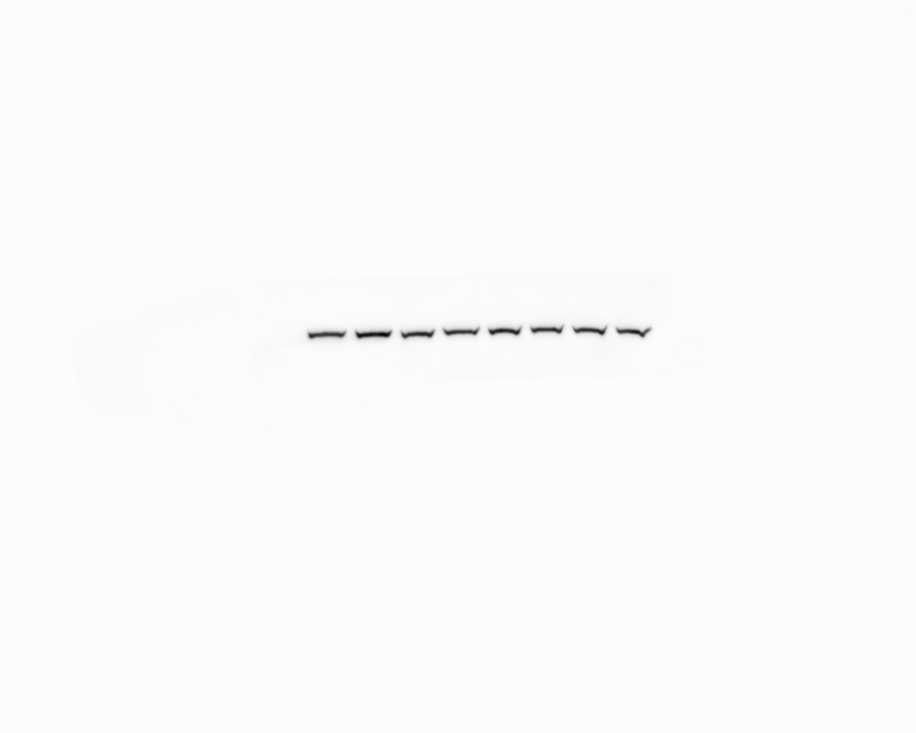

Supplement: Supplementary file 7 [file LSA-2025-03208_SdataF4.zip › Figure_4_Source_Data/tubulin 2023-08-17 13h18m58s(Chemiluminescence).raw16.tif]

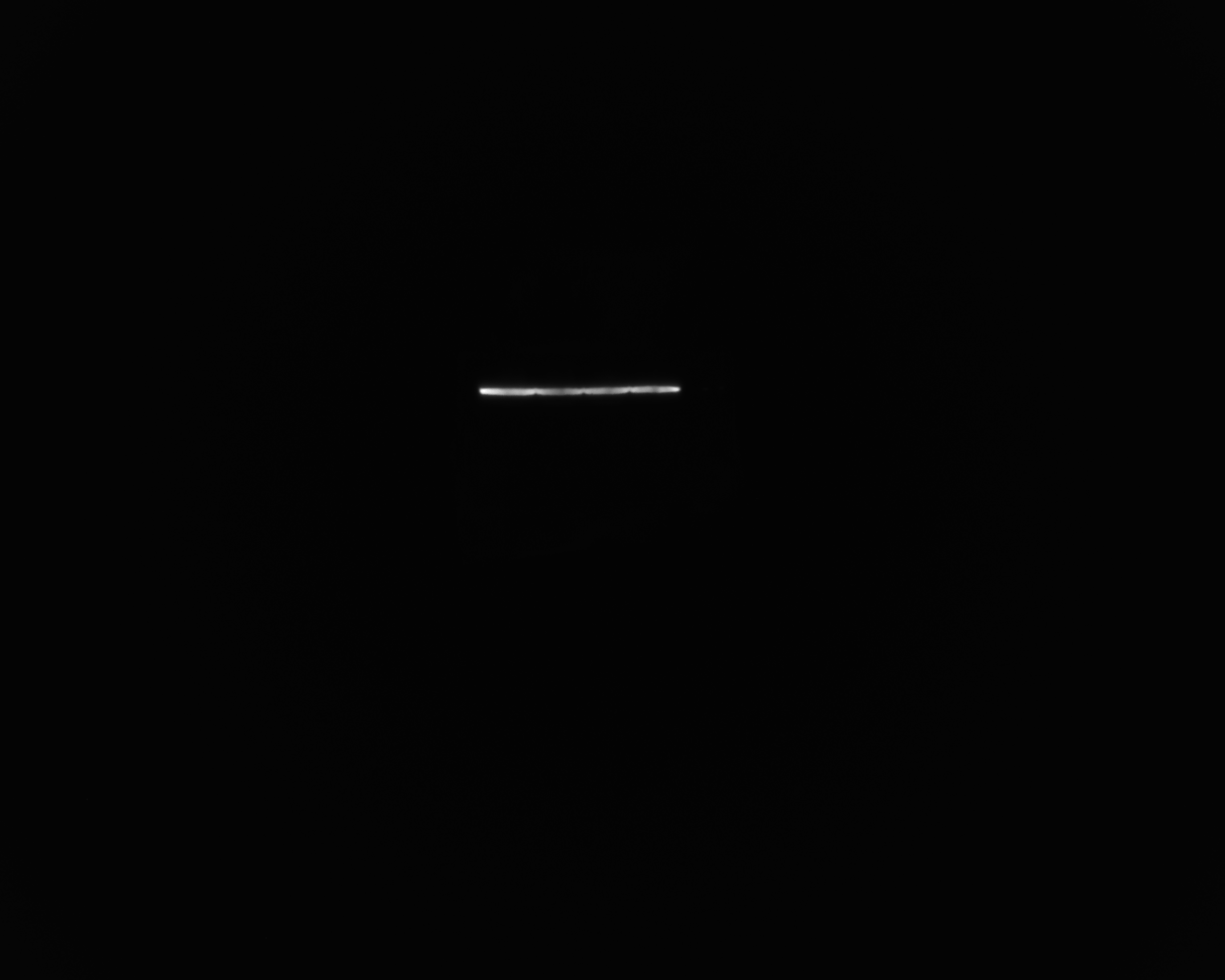

Supplement: Supplementary file 9 [file LSA-2025-03208_SdataF5.zip › Figure_5_Source_Data/H3 2025-03-18 14h23m14s(Chemiluminescence).raw16.tif]

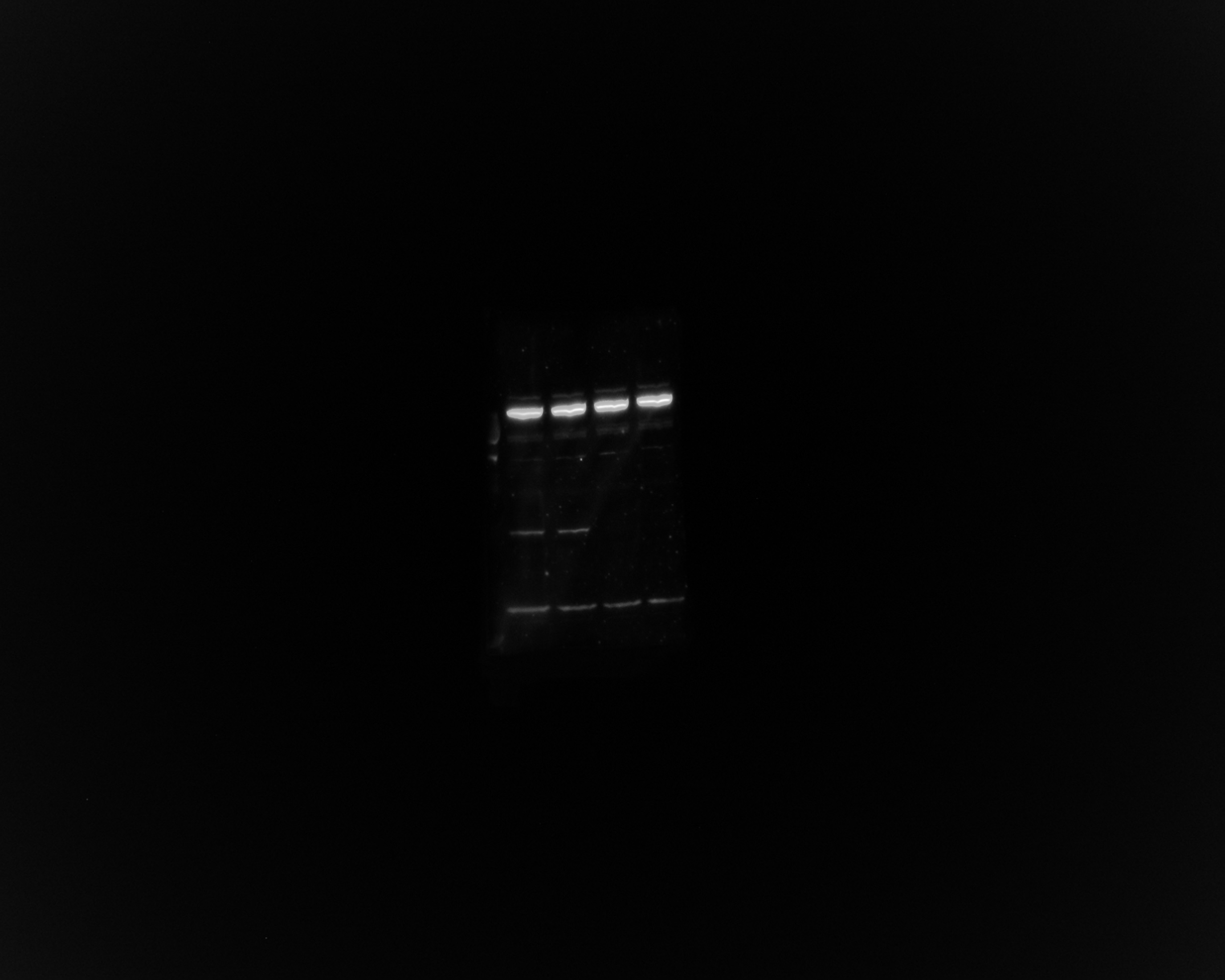

Supplement: Supplementary file 9 [file LSA-2025-03208_SdataF5.zip › Figure_5_Source_Data/nudc whole 2025-03-18 14h27m53s(Chemiluminescence).raw16.tif]

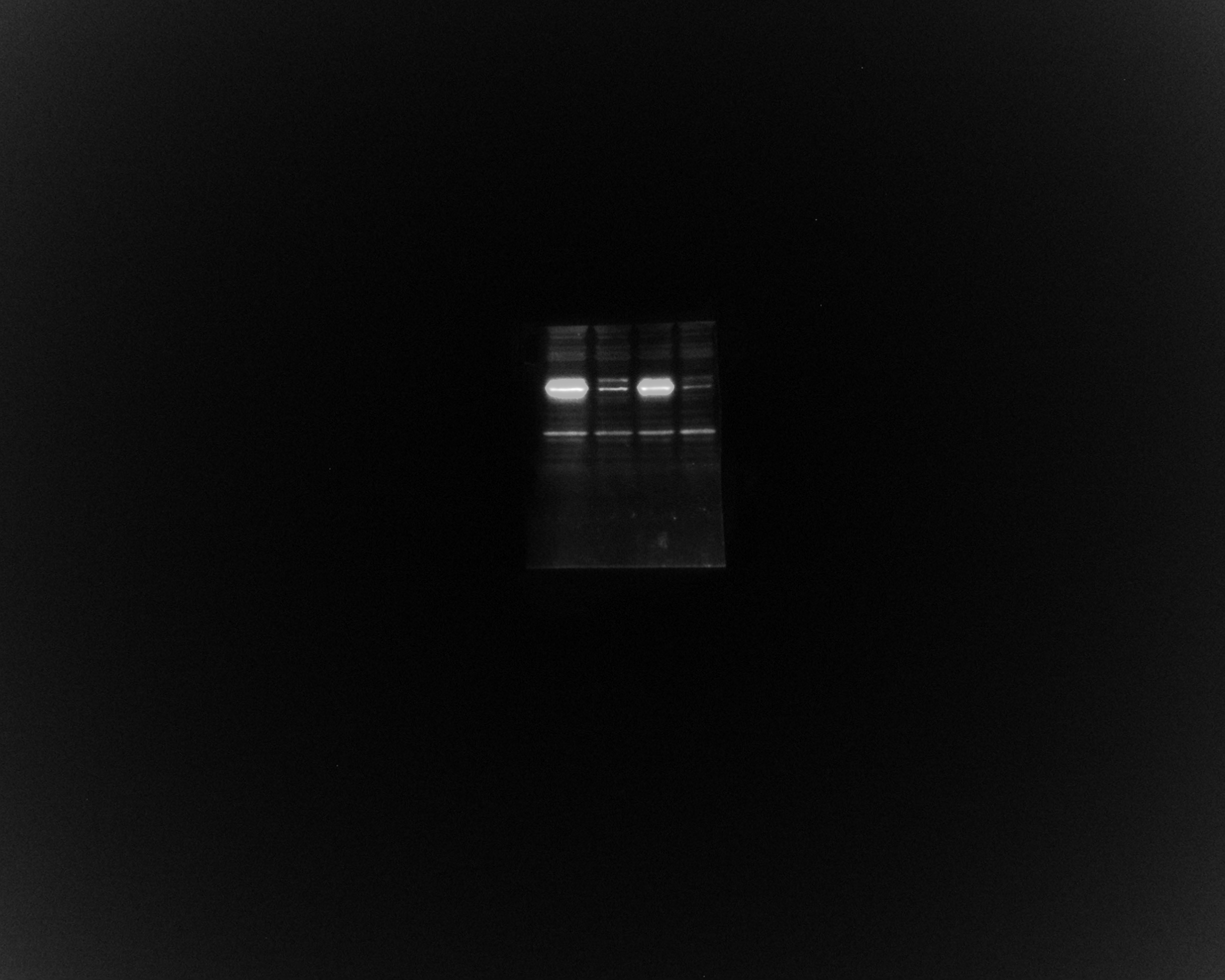

Supplement: Supplementary file 9 [file LSA-2025-03208_SdataF5.zip › Figure_5_Source_Data/psme3 2025-03-14 14h16m30s(Chemiluminescence).raw16.tif]

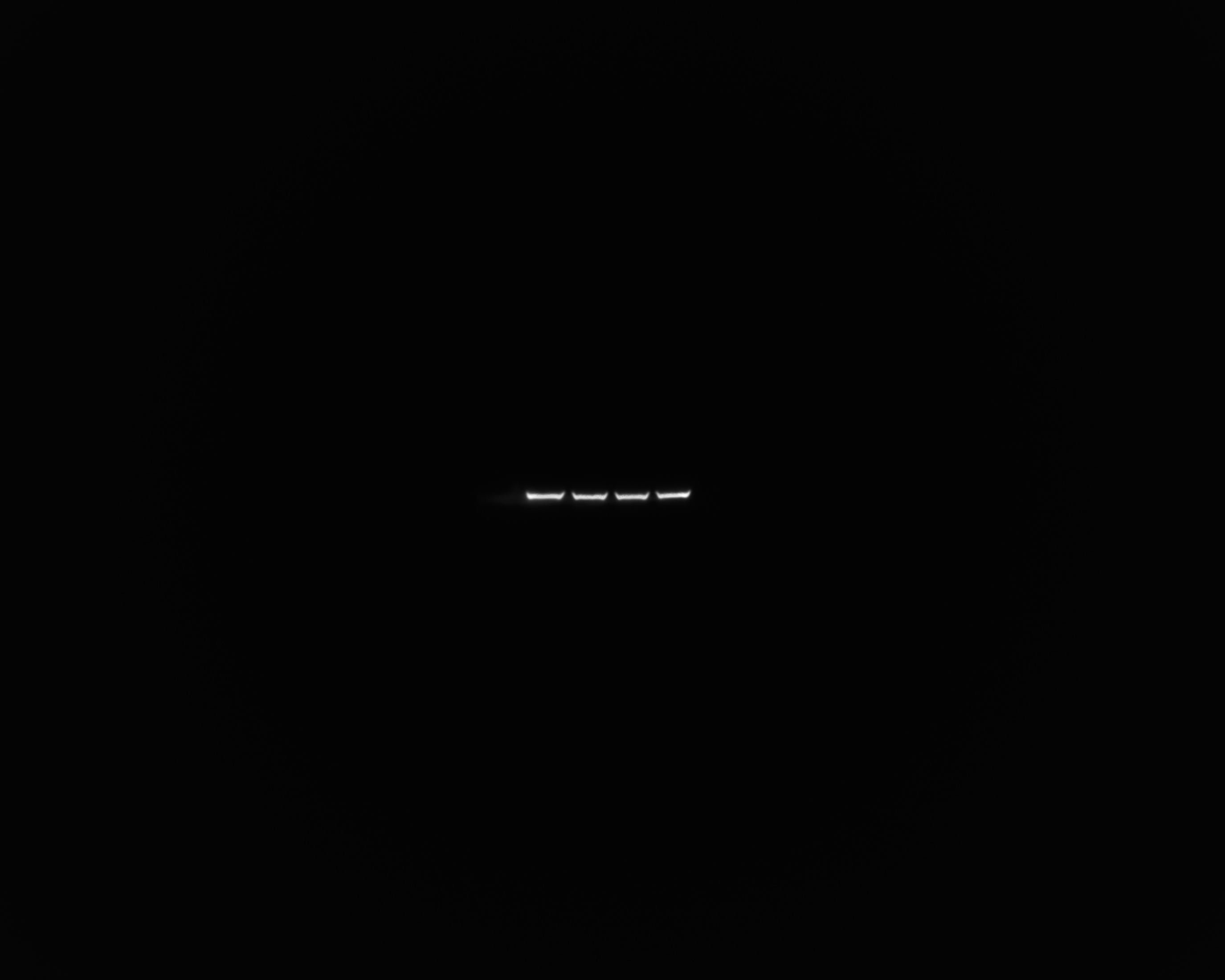

Supplement: Supplementary file 9 [file LSA-2025-03208_SdataF5.zip › Figure_5_Source_Data/tubulin 2025-03-14 14h12m43s(Chemiluminescence).raw16.tif]
